# Supplementing SARS-CoV-2 genomic surveillance with PCR-based variant detection for real-time actionable information, the Netherlands, June to July 2021

**DOI:** 10.2807/1560-7917.ES.2021.26.40.2100921

**Published:** 2021-10-07

**Authors:** Richard Molenkamp, Ewout Fanoy, Leonie Derickx, Theun de Groot, Marcel Jonges, Tjalling Leenstra, Roel Nijhuis, Suzan Pas, Ali Vahidnia, Christian von Wintersdorff, Bert Mulder, Marion Koopmans

**Affiliations:** 1 Department of Viroscience, Erasmus MC, Rotterdam, the Netherlands; 2 Public Health Service Rotterdam-Rijnmond, Rotterdam, the Netherlands; 3 Public Health Service Gelderland-Zuid, Nijmegen, the Netherlands; 4 Department of Clinical Microbiology and Infectious Diseases, Canisius-Wilhelmina Hospital, Nijmegen, the Netherlands; 5 Department of Medical Microbiology and Infection Prevention, Amsterdam University Medical Centers, University of Amsterdam, Amsterdam, the Netherlands; 6 Department of Infectious Diseases, Public Health Service of Amsterdam, Amsterdam, the Netherlands; 7 Laboratory for Medical Microbiology and Medical Immunology, Meander Medical Center, Amersfoort, the Netherlands; 8 Microvida Laboratory for Medical Microbiology, Amphia Hospital, Breda, the Netherlands; 9 Testing Agency, Ministry of Health, Welfare and Sport, Zeist, the Netherlands; 10 Department of Medical Microbiology, Care and Public Health Research Institute (CAPHRI), Maastricht University Medical Centre (MUMC+), Maastricht, the Netherlands

**Keywords:** SARS-CoV-2, COVID-19, Delta variant, VOC, RT-PCR, Public health response

## Abstract

We evaluated routine testing with SARS-CoV-2 Delta variant-specific RT-PCR in regional hospital laboratories in addition to centralised national genomic surveillance in the Netherlands during June and July 2021. The increase of the Delta variant detected by RT-PCR correlated well with data from genomic surveillance and was available ca 2 weeks earlier. This rapid identification of the relative abundance and increase of SARS-CoV-2 variants of concern may have important benefits for implementation of local public health measures.

Assessment of the prevalence of severe acute respiratory syndrome coronavirus 2 (SARS-CoV-2) variants of concern (VOC) by genomic surveillance is impacted by delays. Here, we describe our experiences with routine testing for VOCs by specific RT-PCR in five regional hospital laboratories during the time of emergence of the SARS-CoV-2 Delta VOC (Phylogenetic Assignment of Named Global Outbreak (Pango) lineage designation B.1.617.2) in the Netherlands in June–July 2021. We examined timeliness, as well as trends of RT-PCR typing compared to the centralised national genomic surveillance data, which is based on whole genome sequencing.

## Detection of SARS-CoV-2 variants by specific RT-PCR

National genomic surveillance of SARS-CoV-2 in the Netherlands is performed centrally by a small number of specialised laboratories that contribute sequence data to a national database. However, the time to result for sequencing is impacted by for example delays during sample transport and the complex logistics of the testing landscape (Table 1). RT-PCR assays allow for rapid detection of specific mutations of SARS-CoV-2 using for instance a combination of hydrolysis or hybridisation probes followed by a melting curve analysis [1,2]. When a new VOC is defined and a characteristic set of mutations distinguishing a VOC has been established, variant-specific RT-PCR tests can be developed and implemented in within a week. Variant-specific RT-PCR can be easily implemented in laboratories with RT-PCR capabilities independent of sequencing capabilities and deliver results within a shorter timeframe (Table 1).

**Table 1:** Factors contributing to the turnaround time for identification of SARS-CoV-2 variants of concern, the Netherlands, 2021

Factor	Estimated required time	
	Genomic surveillance	Regional variant-specific RT-PCR
Sample collection	1–8 hours	1–8 hours
Sample transport	2–7 days	NA
Laboratory analysis	2–7 days	2–5 hours
Data interpretation	1–3 days	1–2 hours
Data sharing	1–3 days	1–3 days

NA: not applicable; SARS-CoV-2: severe acute respiratory syndrome coronavirus 2.

Genomic surveillance of SARS-CoV-2 variants is done by a small number of specialised laboratories that contribute whole genome sequences to a central database. Regional variant-specific RT-PCR testing is performed at hospital laboratories.

## Implementation of variant-specific RT-PCR and data collection 

At the beginning of June 2021, the SARS-CoV-2 Delta variant was only sporadically observed in sequence-based national surveillance. To determine the existing capacity for identification of the Delta variant with a specific RT-PCR assay, we sent out a questionnaire to 87 Dutch laboratories that routinely perform SARS-CoV-2 testing. Twelve laboratories reported to have already implemented or have the capability to rapidly implement RT-PCR testing for the Delta variant. Subsequently, data from Delta-specific RT-PCR testing were collected through a weekly survey from week 22 to 27. Weekly reporting was done by five of the 12 laboratories that implemented variant-specific RT-PCR based on either VirSNiP mutation assays (TIB MOLBIOL, Berlin, Germany; four laboratories) or an assay developed in-house (one laboratory) with analytical sensitivities close to the diagnostic SARS-CoV-2 RT-PCR tests. Interpretation of mutation profiles into VOC classification was performed by the local laboratories, which analysed either all SARS-CoV-2-positive samples from test sites and clinical care or took a representative sample selection, depending on the workload. Since the number of SARS-CoV-2-positive tests was dropping considerably at that time, most of the data collected were from all available SARS-CoV-2-positive samples from the indicated weeks.

For four of five laboratories, the contribution of the Delta variant samples to the total number of SARS-CoV-2-positive samples increased exponentially during the 6-week time period. It is however important to note that, from the laboratory using the in-house RT-PCR assay (Figure; L4), only a small number of samples were analysed. The observed increase in the proportion of the Delta variant versus the total as measured by RT-PCR correlated well with the data from the national genomic surveillance (Figure). 

**Figure:**
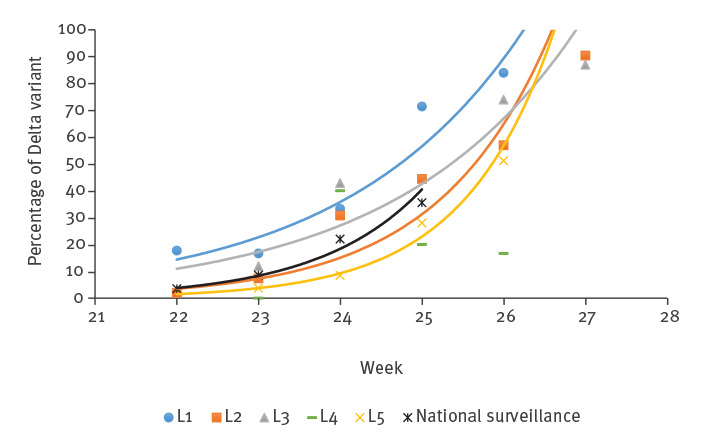
Proportion of the Delta variant among all SARS-CoV-2-positive samples as determined by variant-specific RT-PCR in five regional hospital laboratories compared with data from national surveillance, the Netherlands, June–July 2021 (n = 6)

L: laboratory; SARS-CoV-2: severe acute respiratory syndrome coronavirus 2.

SARS-CoV-2 specific RT-PCR assays performed in five regional hospital laboratories show the proportion of the Delta variant among the total number of SARS-CoV-2-positive samples in a given week. All laboratories used the VirSNiP mutation assay (TIB MOLBIOL, Berlin, Germany) except L4 (green), which used an assay developed in-house. National surveillance data for the Delta variant are generated with whole genome sequencing.

## Comparison of genomic surveillance data with data from variant-specific RT-PCR performed in regional laboratories 

Public reports on the emergence of VOCs based on Dutch national genomic surveillance are updated weekly (https://www.rivm.nl/coronavirus-covid-19/virus/varianten) but, because of long turnaround times, the data are reported 2 to 3 weeks after collection. Because of the shorter turnaround times (Table 2) of variant-specific RT-PCR tests, reports on VOCs could be available within 1 week after collection. In practice, this means that in week 26, the reported national surveillance data are from week 23 while the variant-specific RT-PCR data are from week 25.

**Table 2:** Reporting calendar weeks of variants of concern by genomic surveillance and variant-specific RT-PCR, the Netherlands, June–July 2021

Sample collection (calendar week)	Variant reporting (calendar week)	
	National genomic surveillance	Integrated data from regional variant RT-PCR
22	25	23
23	26	24
24	27	25
25	28	26
26	NA	27
27	NA	28

NA: not applicable.

Genomic surveillance of SARS-CoV-2 variants is done by a small number of specialised laboratories that contribute whole genome sequences to a central database. Regional variant-specific RT-PCR testing is performed at hospital laboratories.

Since sampling was performed during a period with decreasing incidence of SARS-CoV-2 infection in the Netherlands, there was a concern that the lower number of samples in the hospital laboratories would preclude reliable extrapolation of the prevalence of the Delta variant. In addition, as in many cases, no representative sample was taken but instead all SARS-CoV-2-positive samples were analysed. There was concern whether regional testing by variant-specific RT-PCR was more biased towards local outbreaks and clusters. Our analysis shows that, even with these concerns taken into account and with data from only five geographically distributed regional laboratories, there is a good correlation on the trend of emergence of the Delta variant between the national surveillance data and the variant-specific RT-PCR data (Figure). The reliability could potentially be further improved if more regional laboratories would provide data.

## Ethical statement

No ethical approval was required for this study as samples were collected for routine surveillance.

## Discussion and conclusions

Despite the global public health response and the rapid development and roll-out of vaccines, the coronavirus disease (COVID-19) pandemic continues to be a formidable challenge. Given the global inequality in access to vaccines, a likely scenario is that SARS-CoV-2 will continue to spread and cause a sizable disease burden in the years to come. The emergence of VOCs and variants of interest [3-5] brings even more challenges to controlling the pandemic, as new variants may have genetic changes associated with increased transmission in the community and potentially lower vaccine-efficacy. The precise mechanisms for fitness gain remain to be determined, but evidence is accumulating that specific sets of mutations or deletions may be associated with partial escape from immunity, changes in binding affinity to host cells, or increased replication leading to higher viral loads or longer shedding, all potentially contributing to increased likelihood of transmission [6-8].

While much remains to be understood about the role of VOCs on the epidemiology of SARS-CoV-2, there has been well-documented decisive impact on the course of the pandemic from emergence of at least two VOCs. The SARS-CoV-2 Alpha VOC (Pango lineage designation B.1.1.7) was first detected in late 2020 in the United Kingdom (UK) and was associated with rapid expansion and a need for increased public health measures [9]. The emergence of the Delta VOC from May to June 2021 and onward has reversed the downward epidemiological trends in many countries, including those with highest access to vaccines. In many countries in Europe, the emergence of the Delta variant coincided with preparations for relaxing of public health and social measures, which were guided by modelling forecasts. These forecasts take into account the proportion of variant viruses, based on genomic sequencing of a systematic sample of newly diagnosed COVID-19 cases. Given the potential impact of this parameter in model predictions, the turnaround time of these estimates can have an impact on predictions for the near future. Delays in the availability of these data can potentially lead to a delay in the assessment of impact and implementation of control measures. Additionally, the need for laboratories to prioritise resources for the primary diagnosis of COVID-19 while maintaining normal levels of diagnostic capabilities for other diseases, could further interfere with effective genomic surveillance. These aspects are also important in preparedness for future pandemics, but are beyond the scope of this study.

The potential added value of PCR-based variant typing depends on the layout, capacity and turnaround time of the genomic surveillance system. In our study, a clear advantage was that the data from the RT-PCR-based system preceded the publically available national genomic surveillance data by 2 weeks. These additional 2 weeks of actionable time could have implications for local public health response. For instance, after the identification of exponential increase of the Delta variant through RT-PCR analysis in June 2021, the Rotterdam, Amsterdam and Nijmegen regional Public Health Service (PHS) intensified the source and contact tracing. The regional press was informed by the PHS to increase awareness of the public for the continuing relevance of social distancing and need for testing. In the Rotterdam region, testing locations were added to improve surveillance locally, while in the Amsterdam region, the prevalence of the Alpha and Delta VOCs were used as input for predictive models of hospitalisation trends to increase awareness and capacity planning. 

In addition to a faster turnaround time for test results, other benefits of SARS-CoV-2 variant-specific RT-PCR tests are the generally higher sensitivity and lower average costs. In addition, similar RT-PCR tests could be easily implemented to monitor antiviral resistance markers, if antiviral therapies become available in analogue to oseltamivir resistance of the influenza virus [10]. However, since variant-specific RT-PCR tests provide only limited genetic information, genomic surveillance and molecular epidemiology by whole genome sequencing remain important for the identification of new variants and the tracing of clusters or nosocomial transmission.

Remarkably, detection of the Delta VOC in the Netherlands was first thought to be biased by clusters in the bigger cities (e.g. Rotterdam and Amsterdam), comparable with the introduction of the Alpha VOC. However, in Nijmegen, a provincial city, comparable Delta VOC emergence not related to travel was observed. This could be due to the many international business-, trade- and tourist-related connections, allowing easier introduction and spread. Alternatively, initial detection could also be biased by local differences in testing algorithms or higher coverage of surveillance for variants. When resources for genomic surveillance are scarce, a geographically distributed network of sentinel sites, serving as ‘canaries in the coal mine’ for variant-specific RT-PCR screening, could be a viable approach.

It is important to realise that the effectiveness of local response measures depends not only on speed of detection (i.e. early warning), but also on the frequency of VOC introductions, the scale of detected clusters, oversight of potential sources and contacts, compliance of the local population to follow-up advice and, most important, the willingness of policymakers to implement measures. At the time of our analysis and at present, all viral variants are dealt with equally considering basic public health control measures. Therefore, an effective variant-specific response is complex, but buying time in the early phase of spread of a new VOC provides opportunities to increase or adjust control measures that maybe lost otherwise.

## References

[1] Banada P Green R Banik S Chopoorian A Streck D Jones R A simple RT-PCR melting temperature assay to rapidly screen for widely circulating SARS-CoV-2 variants. medRxiv. 2021;JCM0084521. 34288729 10.1128/JCM.00845-21 PMC8451443

[2] Ong DSY Koeleman JGM Vaessen N Breijer S Paltansing S de Man P. Rapid screening method for the detection of SARS-CoV-2 variants of concern. J Clin Virol. 2021;141:104903. 10.1016/j.jcv.2021.104903 34182300 PMC8213512

[3] Harvey WT Carabelli AM Jackson B Gupta RK Thomson EC Harrison EM SARS-CoV-2 variants, spike mutations and immune escape. Nat Rev Microbiol. 2021;19(7):409-24. 10.1038/s41579-021-00573-0 34075212 PMC8167834

[4] World Health Organization (WHO). Tracking SARS-CoV-2 variants. Geneva: WHO. [Accessed: 06 Oct 2021]. Available from: https://www.who.int/en/activities/tracking-SARS-CoV-2-variants

[5] Oude Munnink BB Worp N Nieuwenhuijse DF Sikkema RS Haagmans B Fouchier RAM The next phase of SARS-CoV-2 surveillance: real-time molecular epidemiology. Nat Med. 2021;27(9):1518-24. 10.1038/s41591-021-01472-w 34504335

[6] Kustin T Harel N Finkel U Perchik S Harari S Tahor M Evidence for increased breakthrough rates of SARS-CoV-2 variants of concern in BNT162b2-mRNA-vaccinated individuals. Nat Med. 2021;27(8):1379-84. 10.1038/s41591-021-01413-7 34127854 PMC8363499

[7] Luo CH, Morris CP, Sachithanandham J, Amadi A, Gaston D, Li M, Swanson NJ, Schwartz M, Klein EY, Pekosz A, Mostafa HH. Infection with the SARS-CoV-2 delta variant is associated with higher infectious virus loads compared to the alpha variant in both unvaccinated and vaccinated individuals. medRxiv. 2021 Aug 20:2021.08.15.21262077.

[8] Geers D Shamier MC Bogers S den Hartog G Gommers L Nieuwkoop NN SARS-CoV-2 variants of concern partially escape humoral but not T-cell responses in COVID-19 convalescent donors and vaccines. Sci Immunol. 2021;6(59):eabj1750. 34035118 10.1126/sciimmunol.abj1750 PMC9268159

[9] Davies NG Abbott S Barnard RC Jarvis CI Kucharski AJ Munday JD Estimated transmissibility and impact of SARS-CoV-2 lineage B.1.1.7 in England. Science. 2021;372(6538):eabg3055. 10.1126/science.abg3055 33658326 PMC8128288

[10] van der Vries E Jonges M Herfst S Maaskant J Van der Linden A Guldemeester J Evaluation of a rapid molecular algorithm for detection of pandemic influenza A (H1N1) 2009 virus and screening for a key oseltamivir resistance (H275Y) substitution in neuraminidase. J Clin Virol. 2010;47(1):34-7. 10.1016/j.jcv.2009.09.030 19857993 PMC7185517

